# Hallux valgus correction utilising a modified short scarf osteotomy with a magnesium biodegradable or titanium compression screws – a comparative study of clinical outcomes

**DOI:** 10.1186/s12891-019-2717-7

**Published:** 2019-07-19

**Authors:** Henry Dushan Atkinson, Shahnawaz Khan, Yasha Lashgari, Andreas Ziegler

**Affiliations:** 1 Sports Orthopaedics Research Foundation, 31 Old Broad Street, London, EC2N 1HT UK; 2 North Middlesex University Hospital, Sterling Way, London, N18 1QX UK; 3 University of KwaZulu-Natal, Pietermaritzburg, South Africa

**Keywords:** Hallux valgus, Magnesium, Biodegradable implant, Bioabsorbable implant, Bunion, Scarf osteotomy, Metatarsal displacement osteotomy

## Abstract

**Background:**

Biodegradable implants reduce the likelihood of further surgery for hardware removal and reduce the risks of associated infection and allergy. The purpose of this study is to evaluate the clinical efficacy and determine the comparability of biodegradable magnesium alloy MgYREZr (MAGNEZIX® CS) compression screw fixation compared with standard titanium screw fixation in the surgical treatment of hallux valgus deformity.

**Methods:**

Eleven patients undergoing corrective surgery for hallux valgus utilising biodegradable magnesium screws and a control group of 25 patients undergoing corrective hallux valgus surgery with standard titanium screws were reviewed at a median of 19 months (range 12–30 months). PROM scores (Manchester-Oxford Foot Questionnaire (MOXFQ), Foot and Ankle Outcomes Instrument (FAOI) and the EQ-5D-3 L) were recorded preoperatively and at latest follow-up.

**Results:**

The results between the two groups were broadly similar, with the Magnesium and Titanium patients showing similar patterns in the various domains in the MOXFQ, the FAOI and the EQ-5D-3 L. Most patients reported a near full shoe comfort score, and EQ-5D-3 L scores were significantly improved in both patient groups (with most patients reporting a full score). Foot pain and foot function improved irrespective of the scoring systems and patients in both groups demonstrated significantly improved scores following the surgery (*p* < 0.05). Notably, there were no significant differences when comparing the post-operative scores between the groups for any individual scoring parameter**.** No impairment to quality of life was recorded. There were no intra or post-operative complications. There were no problems encountered through the use of the bioabsorbable screws.

**Conclusion:**

Biodegradable magnesium-based compression screws appeared to be safe in this study and are an effective fixation device in the treatment of hallux valgus deformity with clinical outcomes similar to standard titanium screw fixation.

## Background

Magnesium plates and screws were first used experimentally in the fixation of osteotomies in 1938 [1, 2], though their use remains very limited. MAGNEZIX® CS was the first compression screw to obtain CE marking and was approved for clinical use in 2013. The literature assessing their clinical efficacy is currently limited [3, 4].

The advantages of magnesium-based biodegradable screws have been demonstrated in animal trials [5, 6]. Studies identifying their safe use in humans have also been performed [4, 7–11]. The authors of some human in-vivo studies have suggested that the magnesium potentiates osteoblastic activity and provides a biomechanically stable construct promoting osteo-synthesis [6, 10, 12–16].

Currently, most standard compression screws are composite alloys of non-absorbable materials, typically titanium and steel [17]. The implants that remain in-situ are not completely biologically inert and have the potential to provoke inflammatory reactions and contact allergies well beyond the time of their insertion; nickel and aluminium allergies in particular have been widely reported [18–22]. Thus, when using these metal devices, there is a potential of them having to be removed through further surgery [23].

The risk of infection and inflammatory/allergic responses can be potentially reduced through the use of biodegradable magnesium-based implants [7, 9, 10, 20, 21]. Additionally, magnesium implants reduce artefact on CT scans and have limited noise on magnetic resonance imaging, and therefore do not preclude the use of these imaging modalities, if required [24].

Hardware removal is among the most common of orthopaedics procedures and represents a significant cost to the health economy at large. The risks of anaesthesia, scarring and infection are also increased with recurrent surgery [23, 25]. Foot and ankle implants are more commonly associated with local soft tissue irritation due to their subcutaneous location and their use in weight bearing areas. Biodegradable implants reduce the likelihood of further surgery for hardware removal and reduce the risks of associated infection and allergy [3, 4, 8, 10].

This paper assesses the clinical efficacy of biodegradable magnesium screws compared with standard titanium screws for hallux valgus corrective surgery using a short scarf displacement metatarsal osteotomy.

## Methods

### Study design

Eleven consecutive patients undergoing corrective surgery with MAGNEZIX® CS biodegradable screws for hallux valgus deformity over a 15-month period (February 2016–May 2017) were included in this single centre retrospective study. The indication for hallux valgus surgery was bunion-associated pain with radiographs demonstrating a hallux valgus deformity. Inclusion criteria were patients who were keen to try the biodegradable device in place of a routine titanium screw, and the availability of the magnesium screws on the day of surgery. Patients underwent thorough informed consent and accepted that metal screws might still have been used if the osteotomy fixation was not deemed adequate. Patients also understood that metal staples would be used for an Akin closing wedge proximal phalangeal osteotomy if this was performed. Exclusion criteria were patients with moderate or severe radiological hallux rigidus, those with complex foot deformities requiring surgery involving multiple toes, and those who had clear radiographic signs of osteopenia. The results were compared with a control group of 25 consecutive patients undergoing corrective surgery using standard titanium screws for isolated hallux valgus deformity at the same centre over the same period. All patients were operated only by the senior author in order to minimise variation in the surgical technique. The Institutional review board approved the study protocol, and this study was carried out in accordance with the ethical standards laid down in the 1964 Declaration of Helsinki and its later amendments.

### Surgical technique

Patients were operated in a supine position under general anaesthesia or a local anaesthetic ankle block, with a single dose of intravenous antibiotics and an ankle tourniquet. A medial incision was used, and the capsule was longitudinally incised creating a long dorsal and plantar capsular flap. A small “pocket” was created on the plantar aspect between the capsule and the plantar soft-tissues to facilitate sesamoid balancing (on capsular closure). The bunion was removed using an oscillating saw, and any peripheral osteophytes were removed. An “inside” lateral release was performed though the osteotomy site, releasing the M. adductor hallucis and the lateral sesamoid ligaments.

**Fig. 1:**
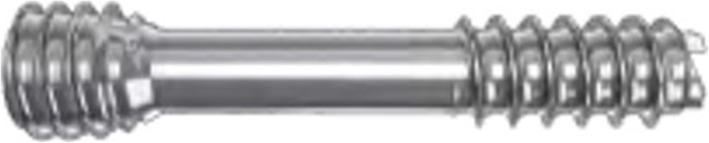
MAGNEZIX® CS 3.2 Compression Screw (Source: Syntellix AG)

A distal 1st metatarsal “short scarf” osteotomy was performed in a routine fashion. The displacement/“shift” was held with a small towel clip and maintained with a 1.1 mm temporary Kirschner wire (K-wire). The correction was verified under mini c-arm fluoroscopic guidance. The K-wire was then overdrilled using a 2.5 mm cannulated drill down to the far plantar cortex. The K-wire was then over drilled using the countersink (In2bones). A MAGNEZIX® CS (3.2 mm diameter) compression screw of an appropriate length was then inserted to maintain the osteotomy position, while also applying compression (Fig. 1). A “vest-over-pants” capsulorrhaphy of the medial capsule was then made.

Bunion corrections in the control patient group were fixed using a single 3.0 mm diameter titanium compression screw, predrilled with a 2 mm drill over a 1 mm K-wire.

Ten of the 11 Magnesium screw patients (91%) and 23 of the 25 Titanium screw patients (92%) also had an Akin closing wedge osteotomy of the 1st proximal phalanx; which the lead author typically performs in the majority of patients for the purposes of toe cosmesis. The Akin osteotomies were fixed using a titanium 8 mm 26° staple.

### Post-operative rehabilitation

Patients were mobilised fully weightbearing in a heel-wedge shoe for 6 weeks, strictly elevating the foot for the first 7 days. Patients were encouraged to gently manipulate their great toes with dorsal and plantar stretches from 7 days. Skin sutures were removed after 2 weeks. After 6 weeks patients were clinically assessed, radiographs were taken, and patients were then mobilised in normal flat footwear. There were no adverse radiographic findings seen in any patient, and all patients were routinely discharged 6 weeks post-operatively. No patient required physiotherapy for toe stiffness. The authors could not justify routinely repeating radiographs after 3, 6, and 12 months given the unnecessary patient exposure to radiation; unless there was a clinical indication to do so. Previous small studies have looked at the radiological (X-ray) and MRI biodegradation of magnesium-based screws in the treatment of hallux valgus and have found that the screws completely degrade by 2–3 years after surgery without any sequelae [8–11].

Patients were contacted at a median of 19 months post-operatively, and at a minimum of 12 months (range 12–30 months) for the purposes of this study. Patients were interviewed by phone and all 36 patients returned their fully completed questionnaires.

**Fig. 2:**
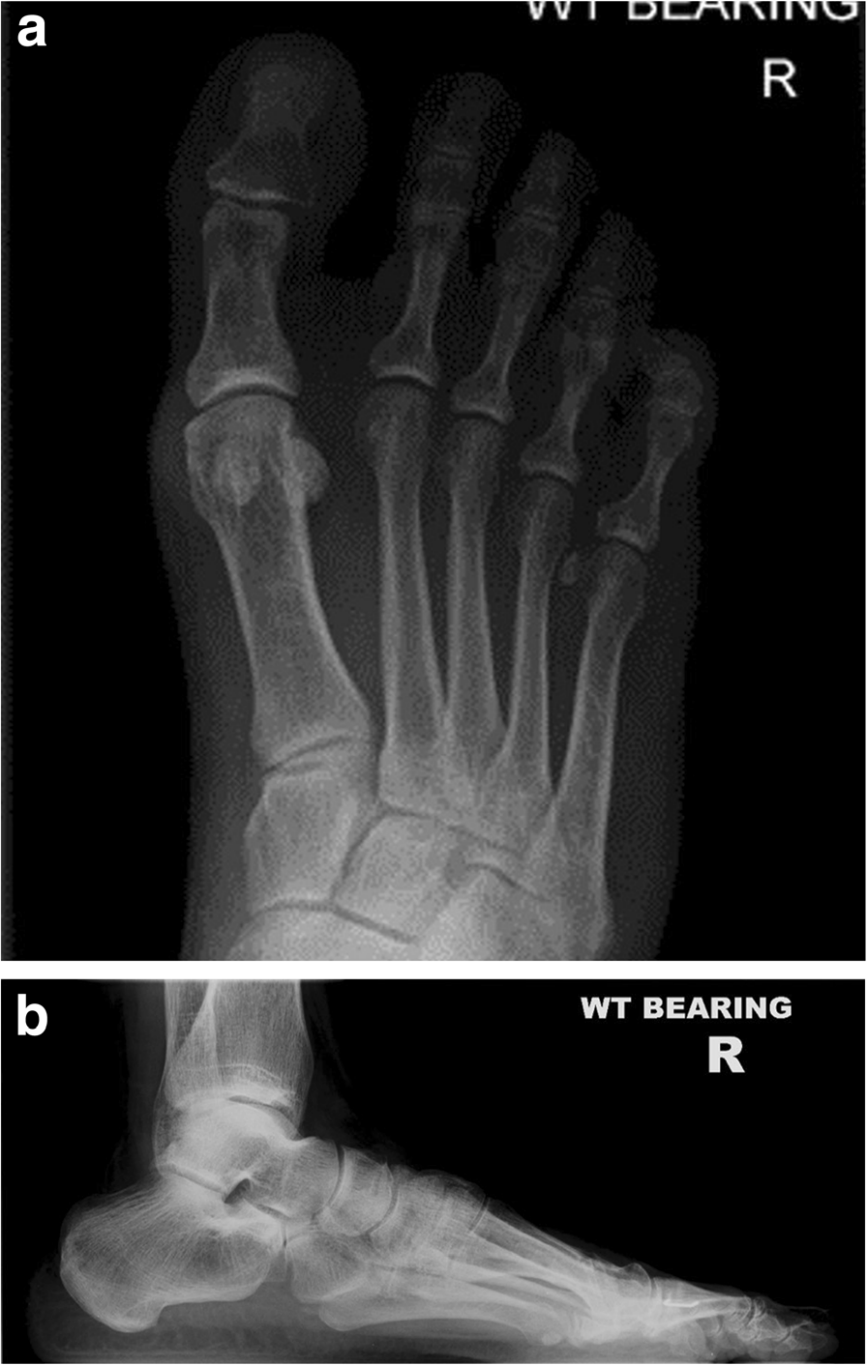
**a** and **b** Pre-operative AP and lateral radiographs

**Fig. 3:**
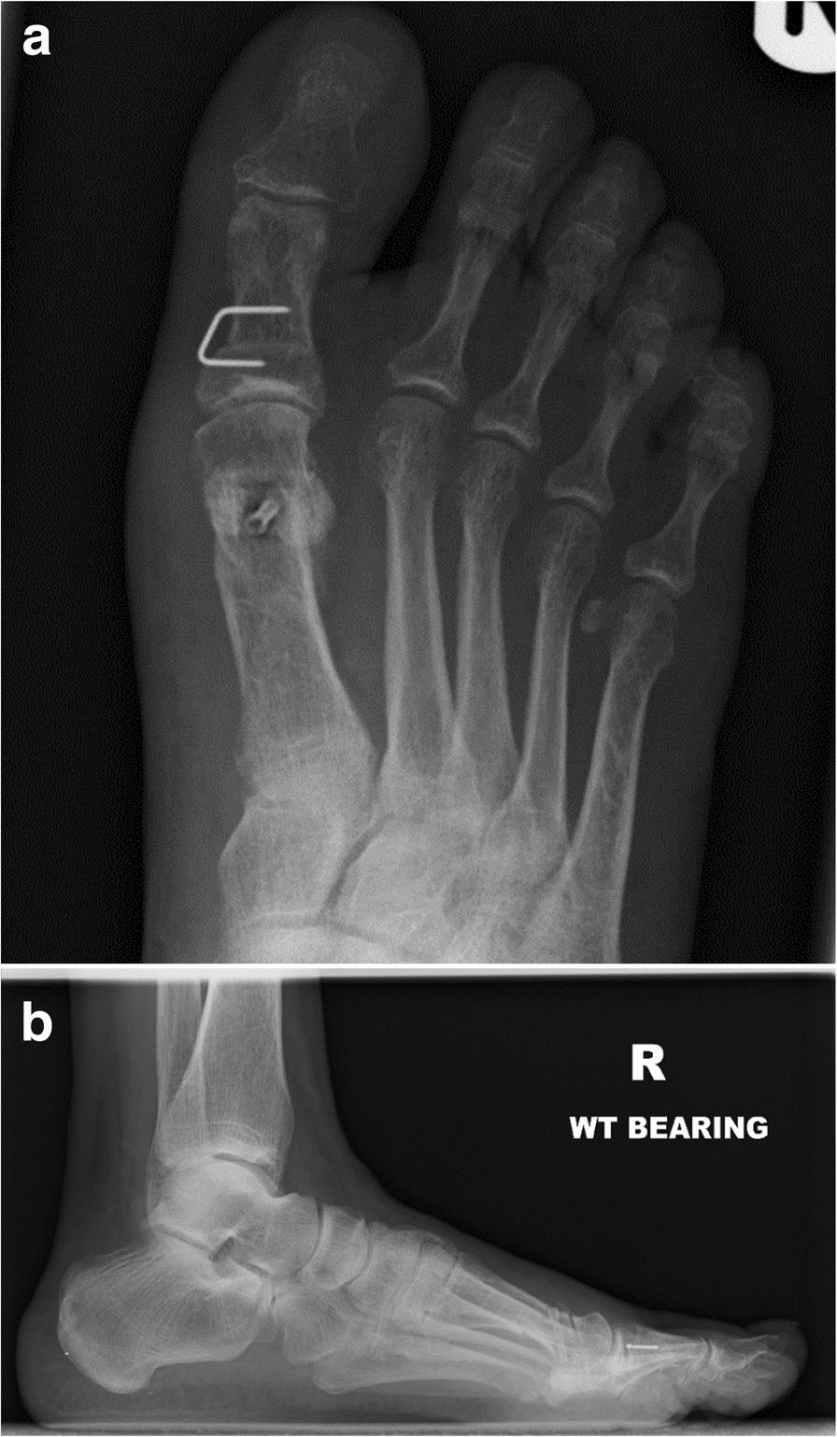
**a** and **b** Post-operative AP and lateral radiographs taken at six weeks follow up

**Fig. 4:**
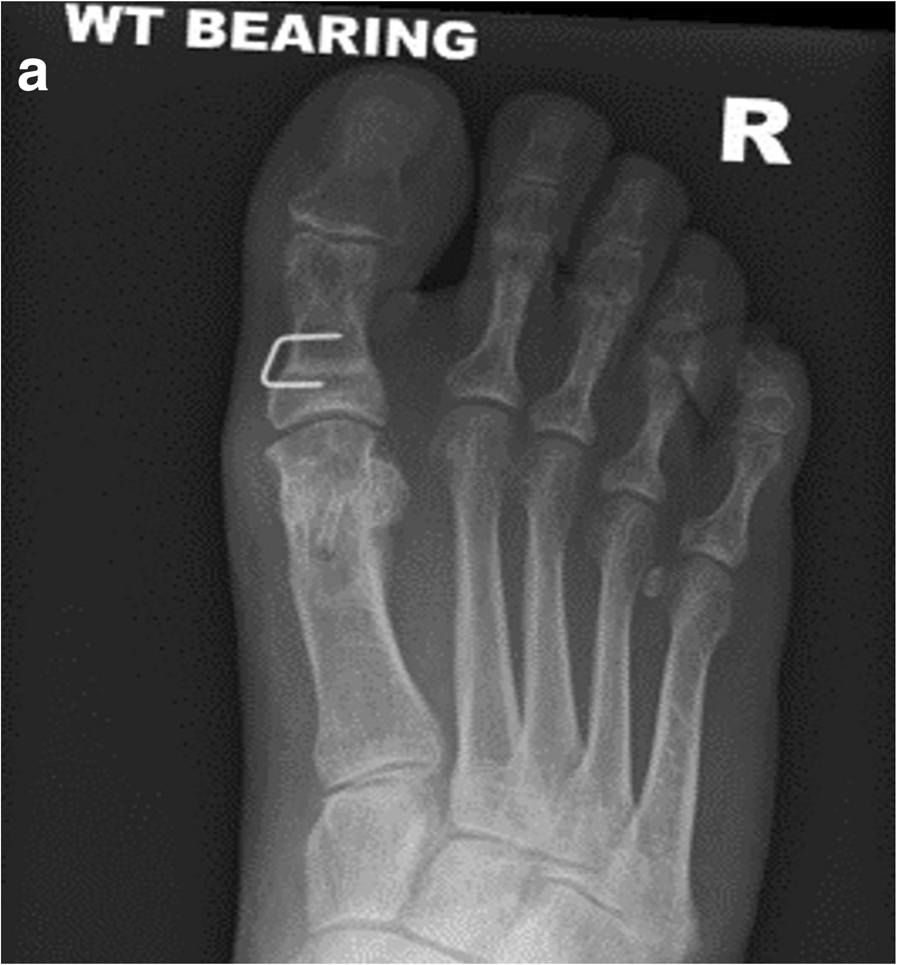
**a** and **b** Post-operative AP and lateral radiographs taken at 12 months follow up

One MAGNEZIX® patient who was re-referred back to our unit for other unrelated reasons had repeat radiographs taken of their operated foot at 12 months following the index surgery. This patient’s radiographs are presented in Figs. 2, 3 and 4 and show the typical radiographic features of magnesium-based biodegradable screws.

### Scoring systems

Validated scoring instruments were used to assess patient satisfaction and clinical efficacy of the surgery. The Manchester–Oxford Foot Questionnaire, and the Foot and Ankle Outcome Instrument were used to assess foot function, and the EQ-5D index was used to determine quality of life parameters [26–30]. Scores were recorded in accordance with published surveys and were performed by an independent observer. All patients gave their written informed consent to be included in the study. A patient information leaflet was provided.

### Statistics

The Wilcoxon test for paired data was used for analysing non-parametric data, and a significance level of 0.05 was deemed significant. Hodges-Lehmann confidence intervals were calculated for preoperative – postoperative values. Statistical calculations were performed using R (r-project.org).

## Results

Of the 11 MAGNEZIX® patients 9 were female (82%). Mean age at the time of surgery was 38 years (range (25–51 years). Right foot/left foot ratio was 6:5. Four patients had a mild (15–20 degree) and 7 a moderate (21–40 degree) hallux valgus deformity. Mean hallux valgus angle (HVA) was 28.7 degrees ±5.2 degrees. All patients were operated on a day-case basis. The mean operative time was 35 min. There were no intra-operative or post-operative complications noted. There were no wound-healing issues, no infections and no clinical adverse events to report.

Of the 25 titanium patients 23 were female (92%). Mean age at the time of surgery was 41 years (range (26–72 years). Right foot/left foot ratio was 16:9. Ten patients had a mild (15–20 degree) and 15 a moderate (21–40 degree) hallux valgus deformity. Mean HVA was 27.3 degrees ±7.0 degrees All patients were operated on a day-case basis. The mean operative time was 34 min. There were no intra-operative or post-operative complications noted. There were no wound-healing issues, no infections and no adverse events to report. No patient from either group required revision surgery or surgery to remove hardware.

Post-operative imaging at 6 weeks did not identify any loss of correction compared with the intra-operative imaging in any patient. Radiological assessment did not identify any screw loosening or migration. The MAGNEZIX® implant is less radiopaque than a titanium screw. Figs. 2, 3 and 4 demonstrate the images from one of the patients; 1a) pre-operative AP and 1b) lateral radiograph, 2a) post-operative AP and 2B0 lateral radiographs at the 6 week review (demonstrating the hydrogen gas bubble), 3a) post-operative AP and 3b) lateral radiograph at 12 month review.

The MOXFQ score parameters for walking/standing, foot pain and social interaction (33.44,36.36,42.61 respectively) all improved significantly following surgery (6.82,10.45, 1.14) in the MAGNEZIX® patient group; with foot pain and social interaction showing the greatest levels of improvement. In terms of foot function, the FAOI scale demonstrated a marked improvement from a mean of 82.82 to 96.89 (*p* < 0.004) in the MAGNEZIX® patient group (Table 1). Similar patterns were observed in the control group, where all the parameters also significantly improved. In the titanium patients, the MOXFQ score parameters for walking/standing, foot pain and social interaction (20.29.26.6,38.5 respectively) all improved significantly following surgery (7.14, 5.2,4.75) with foot pain and social interaction similarly showing the greatest levels of improvement. The FAOI scale also significantly improved from a mean of 84.82 to 95.53 (*p* < 0.001) (Table 2).

**Table 1:** Preoperative and postoperative score values for each dimension of the Manchester-Oxford Foot Questionnaire (MOXFQ), the Foot and Ankle Outcome Index (FAOI) and quality of life (EQ-5D-3 L) in the MAGNEZIX® patient cohort. *N* = 11

Score	Pre-OP	Post-OP	Median diff pre – post	*P*-value
MOXFQ				
Walking/standing	33.44 ± 11.21	6.82 ± 8.06	26.79 (17.86–33.93)	0.004
Foot pain	36.36 ± 9.51	10.45 ± 9.34	25.00 (20.00–32.50)	0.004
Social interaction	42.61 ± 8.30	1.14 ± 2.53	40.63 (34.38–46.88)	0.004
Index	37.47 ± 8.47	6.14 ± 6.01	31.35 (25.95–36.07)	0.004
FAOI				
Core scale	82.82 ± 4.64	96.89 ± 2.91	−14.04 (−16.88 – −11.25)	0.004
Core scale mormative	41.62 ± 3.77	53.03 ± 2.36	−11.39 (−13.69 – − 9.12)	0.004
Shoe comfort scale	43.64 ± 20.38	82.73 ± 24.12	−38.32 (− 55.00 – −22.50)	0.004
Shoe norm	39.75 ± 6.91	53.00 ± 8.18	− 13.27 (− 18.64 – − 7.63)	0.004
EQ-5D-3 L Index	0.83 ± 0.11	1.00 ± 0.00	− 0.22 (− 0.26 – − 0.20)	0.012
VAS	80.00 ± 12.38	88.91 ± 11.84	−9.00 (− 15.00 – − 4.00)	0.006

Displayed are means ± standard deviations; *Pre-op* Preoperative, *Post-op* Postoperative; Median diff pre – post: difference of medians plus 95% Hodges-Lehmann confidence interval

**Table 2:** Preoperative and postoperative score values for each dimension of the Manchester-Oxford Foot Questionnaire (MOXFQ), the Foot and Ankle Outcome Index (FAOI) and quality of life (EQ-5D-3 L) in the titanium patient cohort. *N* = 25

Score	Pre-OP	Post-OP	Median diff pre – post	*P*-value
MOXFQ				
Walking/standing	20.29 ± 13.35	7.14 ± 8.38	14.29 (10.71–17.86)	0.001
Foot pain	26.60 ± 13.05	5.2 ± 6.84	20.00 (17.50–25.00)	0.001
Social interaction	38.50 ± 17.56	4.75 ± 10.09	34.38 (28.12–40.63)	0.001
Index	28.46 ± 13.48	5.70 ± 7.56	22.80 (19.40–26.37)	0.001
FAOI				
Core scale	84.82 ± 5.25	95.53 ± 2.25	− 10.71 (− 12.50 – − 8.75)	0.001
Core scale normative	43.25 ± 4.26	51.93 ± 1.82	−8.69 (− 10.14 – − 7.10)	0.001
Shoe comfort scale	41.60 ± 18.41	83.20 ± 22.12	−40.00 (− 55.00 – − 22.50)	0.001
Shoe normative	39.06 ± 6.24	53.16 ± 7.50	− 13.56 (− 16.95 – − 11.02)	0.001
EQ-5D-3 L				
Index	0.86 ± 0.24	0.96 ± 0.12	− 0.27 (− 0.62 – − 0.19)	0.014
VAS	81.52 ± 11.82	90.68 ± 9.45	− 10.00 (− 12.50 – − 6.50)	0.001

Displayed are means ± standard deviations; *Pre-op* Preoperative, *Post-op* Postoperative; Median diff pre – post: difference of medians plus 95% Hodges-Lehmann confidence interval

When comparing the magnesium and titanium patient groups, the clinical scores were broadly similar. Most patients reported a near full shoe comfort score, and EQ-5D-3 L scores were significantly improved in both patient groups (with most patients reporting a full score).

Foot pain and foot function improved irrespective of the scoring systems and patients in both groups demonstrated significantly improved scores following the surgery (*p* < 0.05). Notably, there were no significant differences when comparing the post-operative scores between the groups for any individual scoring parameter (Table 3).

**Table 3:** A comparison of magnesium and titanium patient groups pre-surgery (Pre), post-surgery (Post), and the changes between pre- and post-surgery (Post-pre) for each dimension and index for the Manchester-Oxford Foot Questionnaire (MOXFQ), the Foot and Ankle Outcome Index (FAOI) and quality of life (EQ-5D-3 L)

Score / domain	Comparison	Med diff pre – post	*P*
MOXFQ			
Walking/standing	Pre	−14.29 (− 25.00 – − 3.57)	0.008
	Post	0.00 (−3.57–3.57)	0.874
	Post-pre	14.28 (7.14–21.43)	0.001
Foot pain	Pre	−10.00 (−20.00 – − 5.00)	0.015
	Post	-5.00 (−15.00–0.00)	0.088
	Post-pre	5.00 (0.00–10.00)	0.114
Social interaction	Pre	−6.25 (−18.75–6.25)	0.283
	Post	0.00 (0.00–0.00)	0.416
	Post-pre	6.25 (0.00–18.75)	0.068
Index	Pre	−11.13 (− 19.76 – −0.77)	0.036
	Post	−0.48 (−6.19–2.38)	0.653
	Post-pre	8.63 (3.15–14.17)	0.008
FAOI			
Core scale	Pre	2.42 (−1.75 – −6.17)	0.229
	Post	−1.75 (−3.25–0.50)	0.099
	Post-pre	−3.42 (−6.67 – −0.08)	0.036
Core scale normative	Pre	1.96 (−1.42–5.00)	0.229
	Post	−1.42 (−2.64–0.41)	0.099
	Post-pre	−2.77 (−5.41 – −0.07)	0.038
Shoe comfort scale	Pre	0.00 (−15.00–10.00)	0.943
	Post	0.00 (−10.00–10.00)	0.939
	Post-pre	0.00 (−10.00–25.00)	0.931
Shoe normative	Pre	0.00 (−5.08–3.39)	0.943
	Post	0.00 (−3.39–3.39)	0.939
	Post-pre	0.00 (−3.39–8.47)	0.904
EQ-5D-3 L			
Index	Pre	0.052 (0.00–0.20)	0.233
	Post	0.00 (0.00–0.00)	0.252
	Post-pre	−0.16 (−0.20–0.00)	0.079
VAS	Pre	3.00 (−7.00–11.00)	0.667
	Post	0.00 (−5.00–10.00)	0.931
	Post-pre	0.00 (−5.00–6.00)	0.877

Med diff: difference of medians plus 95% Hodges-Lehmann confidence interval; *p p*-value from Wilcoxon test for independent samples

The magnesium patients did however have significantly greater improvement in several of the “post-pre” scoring parameters in the MOXFQ; this was largely due the magnesium patients having suffered significantly worse levels of pre-operative symptoms in these specific parameters, while achieving similar post-operative scores. These included the MOXFQ walking/standing post-pre scores, and the MOXFQ post-pre index scores. Pre-operative MOXFQ foot pain was also greater in the magnesium patients. The authors highlight that this larger improvement should not be interpreted as demonstrating any superiority of Magnesium fixation, as the final clinical scores are similar and not significantly different between the two patient groups.

## Discussion

This small study has demonstrated that corrective surgery using a modified short scarf osteotomy is clinically effective for isolated minimal and moderate isolated bunion deformities with patients improving significantly in the MOXFQ, FAOI, and EQ-5D-3 L scoring modalities. This study has also demonstrated that the biodegradable MAGNEZIX® CS compression screw is clinically effective, and it was safely used for the fixation of a short scarf displacement metatarsal osteotomy in hallux valgus corrective surgery. The MAGNEZIX® CS compression screw has shown similar results to the standard titanium fixation screws used in the control group, with clinical results also comparable with the literature [4, 8–11, 16]. The patient reported outcomes in this study are also comparable to existing data relating MAGNEZIX® screws; these other MAGNEZIX® studies also found that patients suffered no long-term pain symptoms, no procedural infections, no loss of fixation position and with high levels of satisfaction [7–10, 16, 31, 32].

This implant offers similar fixation to conventional titanium screws, with the benefit of not requiring hardware removal [7, 16]. Routine implant removal represents a significant burden on the health economy and carries with it the risks of infection scarring and possible neurovascular injury [23, 25, 33]. Magnesium-based screws confer the advantage of biodegrading via corrosion rather than conventional implants that hydrolyse [4, 6, 10, 34, 35]. Corrosion reduces the inflammatory response associated with screw absorption and is less irritant to surrounding tissues, thus minimising osteolysis [3, 4, 10]. Histological analyses performed in animal studies demonstrated the time course over which the implants degrade [2, 6, 36]. In one study at 12 months magnesium screws had completely reabsorbed replaced by bony ingrowth and potassium crystals; importantly these potassium crystals are biologically inert and do not affect bone formation suggesting that once fully degraded the bone micro architecture ostensibly returns to normal [6].

During the natural corrosion process of magnesium, hydrogen is created, and this forms a temporary radiolucency, either seen around the magnesium screw or in the neighbouring dorsal soft-tissues. This resolves of its own accord without sequelae [10]. Corrosion analysis indicates that MAGNEZIX implants hold stability for the initial 6–12 weeks and are completely corroded within 2–3 years [4, 9, 12, 16].

There were no complications or intraoperative technical problems encountered, however, the authors would like to stress that the material characteristics of these biodegradable screws are different to those of the conventional metal screws. There is a learning curve in their use. The screws are not self-drilling or self-tapping, and the countersink drill must be used in every case; though pre-tapping is not necessary prior to screw insertion. The MAGNEZIX® CS compression screws have a Young’s modulus similar to natural bone, but lower than titanium screws. Hence, the screw heads are more prone to fatigue or fracture on insertion (compared with the conventional metal bi-compression screws), particularly if large torque is applied. These screws should be inserted with care and with reduced torque to prevent sheering of the implant. Once familiar we found that the handling of these screws was not greatly different to the metal screw equivalents, and once inserted the compression and stability of the osteotomy appeared clinically comparable to that which we see with titanium implants.

Though very encouraging, this is a small cases series and prior to the widespread adoption of this device it remains imperative that equivalence is demonstrated in comparison to the current gold standard; and the authors would recommend and support a multicentre randomised prospective trial assessing its efficacy.

## Conclusion

The MAGNEZIX® CS compression screw is a biodegradable implant made of a magnesium alloy (MgYREZr). Fixation of displacement 1st metatarsal osteotomies in the surgical management of hallux valgus with the MAGNEZIX® CS metal biodegradable compression screw provides adequate fixation with good patient-reported outcomes comparable with more conventional metal fixation devices.

## Data Availability

All the data analyses from this study are included in this published article in the Tables 1, 2, 3. The raw datasets generated are not publicly available due a lack of an appropriate online repository but are available from the corresponding author on reasonable request.

## Abbreviations

CS: Compression screw
CT: Computed tomography
EQ-5D-3 L: EuroQol Group generic health status
FAOI: Foot and Ankle Outcomes Instrument
MOXFQ: Manchester-Oxford Foot Questionnaire
PROM: Patient Rated Outcome Measure
